# *In vitro* activity of zoliflodacin against *Neisseria gonorrhoeae* collected in the United States from 2020 to 2022

**DOI:** 10.1128/aac.00527-26

**Published:** 2026-08-03

**Authors:** Sarah M. McLeod, Samir H. Moussa, Meredith Hackel, Carmen Au, Pierre Daram

**Affiliations:** 1 Innoviva Specialty Therapeutics, Inc., an affiliate of Entasis Therapeutics Inc., Waltham, Massachusetts, USA; 2 IHMA, Inc., Schaumburg, Illinois, USA; 3 Global Antibiotic R&D Partnership, Geneva, Switzerland; Shionogi Inc., Florham Park, New Jersey, USA

**Keywords:** zoliflodacin, gonorrhea, antibiotic resistance, *Neisseria gonorrhoeae*, DNA gyrase

## Abstract

Zoliflodacin is a first-in-class spiropyrimidinetrione, oral, single-dose antibacterial recently approved by the US FDA for treatment of uncomplicated urogenital gonorrhea. Isolates of *Neisseria gonorrhoeae* (*n* = 322) collected in the United States in 2020–2022 by the Centers for Disease Control and Prevention, as well as two ceftriaxone-resistant isolates, were tested for susceptibility to zoliflodacin. All isolates were susceptible to zoliflodacin with MIC_50/90_ values of 0.12 µg/mL. Zoliflodacin activity was consistent over time and against ceftriaxone-, ciprofloxacin-, and tetracycline-resistant and azithromycin-non-susceptible isolates.

## INTRODUCTION

Gonorrhea, caused by *Neisseria gonorrhoeae*, was the second most common nationally notifiable sexually transmitted infection in the United States in 2022 (1). *N. gonorrhoeae* can infect a variety of mucosal surfaces, including the urethra, endocervix, pharynx, conjunctiva, and rectum (2). Over several decades, treatment of gonorrhea has been complicated by the ability of *N. gonorrhoeae* to rapidly develop resistance to a range of antibiotic classes (3). There is particular concern about rising rates of resistance to current standard-of-care antibiotics for the treatment of gonorrhea. The World Health Organization (WHO) Enhanced Gonococcal Antimicrobial Surveillance Program found that, among *N. gonorrhoeae* collected in 2024 from 12 countries, 5% of isolates had ceftriaxone MICs > 0.12 µg/mL, 11% had cefixime MICs > 0.12 µg/mL, and 4% had azithromycin MICs > 1 µg/mL (4). The development of resistance to all drug classes previously used as treatment for gonorrhea has led the Centers for Disease Control and Prevention (CDC) and the WHO to list *N. gonorrhoeae* as a priority pathogen for which novel agents are urgently needed (5, 6).

In December 2025, zoliflodacin was approved by the US FDA for the treatment of uncomplicated urogenital gonorrhea (7). Zoliflodacin is a first-in-class spiropyrimidinetrione, oral, single-dose antibacterial, which inhibits DNA biosynthesis. Zoliflodacin has a unique mode of action, primarily targeting the GyrB subunit of DNA gyrase, differentiating zoliflodacin from fluoroquinolones, which also inhibit DNA gyrase but primarily interact with the GyrA subunit (8). In a recent, global, randomized phase 3 clinical trial, zoliflodacin was shown to be non-inferior to a combination therapy of ceftriaxone plus azithromycin for microbiological cure of urogenital gonorrhea (9).

This study characterized the *in vitro* activity of zoliflodacin against US *N. gonorrhoeae* isolates provided by the CDC, which were collected as part of the Gonococcal Isolate Surveillance Project (10). A total of 322 *N. gonorrhoeae* isolates were collected from sexually transmitted disease clinics across the United States during 2020 (*n* = 99), 2021 (*n* = 100), and 2022 (*n* = 123) from males (*n* = 287) and females (*n* = 26) and 9 unspecified. The *N. gonorrhoeae* strains were isolated from urogenital (*n* = 312), pharyngeal (*n* = 7), and rectal (*n* = 3) sites of infection. Species identification was confirmed by matrix-assisted laser desorption/ionization time-of-flight, and isolates were assayed by agar dilution using Clinical and Laboratory Standards Institute (CLSI) methodologies for susceptibility to zoliflodacin and eight other antibiotics used to treat gonorrhea (11).

Among the 322 *N. gonorrhoeae* tested, all isolates were susceptible to ceftriaxone according to the CLSI susceptibility breakpoint (MICs ≤ 0.12 µg/mL) (12). The MIC_50/90_ of ceftriaxone was 0.008/0.015 µg/mL with a MIC range of ≤0.002 to 0.06 µg/mL. Most of the isolates were also susceptible to azithromycin (96.3% susceptible). In contrast, only 63.4% and 9.9% of isolates were susceptible to ciprofloxacin and tetracycline, respectively. Results for each year between 2020 and 2022 were similar (Table 1).

**TABLE 1:** *In vitro* activities of zoliflodacin and comparator antibacterial agents tested against isolates of *Neisseria gonorrhoeae* collected in the United States from 2020 to 2022^*c*^

Year collected (no. of isolates)	Antibacterial agent	MIC (µg/mL)			MIC interpretation^*a*^		
		MIC_50_	MIC_90_	Range	%S	%I	%R
All (322)	Zoliflodacin	0.12	0.12	≤0.008–0.25	100	NA	NA
	Azithromycin	0.12	1	≤0.06 to >8	96.3	NA	NA
	Cefixime	0.015	0.03	≤0.002–0.12	97.8	2.2	0
	Ceftriaxone	0.008	0.015	≤0.002–0.06	100	0	0
	Ciprofloxacin	0.004	>2	0.001 to >2	63.4	1.6	35.1
	Gentamicin	8	8	1–16	NA	NA	NA
	Penicillin	0.5	>2	≤0.12 to >2	NA	84.8^*b*^	15.2
	Spectinomycin	16	32	≤4–32	100	0	0
	Tetracycline	1	>4	≤0.12 to >4	9.9	73.6	16.4
2020 (99)	Zoliflodacin	0.06	0.12	≤0.008–0.25	100	NA	NA
	Azithromycin	0.25	1	≤0.06 to >8	92.9	NA	NA
	Cefixime	0.015	0.03	≤0.002–0.12	99.0	1.0	0
	Ceftriaxone	0.008	0.015	≤0.002–0.06	100	0	0
	Ciprofloxacin	0.004	>2	0.002 to >2	59.6	4.0	36.4
	Gentamicin	8	8	4–16	NA	NA	NA
	Penicillin	0.25	>2	≤0.12 to >2	NA	76.8^*b*^	23.2
	Spectinomycin	16	16	≤4–32	100	0	0
	Tetracycline	1	>4	≤0.12 to >4	10.1	70.7	19.2
2021 (100)	Zoliflodacin	0.06	0.12	≤0.008–0.12	100	NA	NA
	Azithromycin	0.25	1	≤0.06–2	98.0	NA	NA
	Cefixime	0.015	0.03	≤0.002–0.12	98.0	2.0	0
	Ceftriaxone	0.008	0.015	≤0.002–0.03	100	0	0
	Ciprofloxacin	0.004	>2	0.002 to >2	67.0	0	33.0
	Gentamicin	4	8	2–8	NA	NA	NA
	Penicillin	0.5	2	≤0.12 to >2	NA	87.0^*b*^	13.0
	Spectinomycin	16	32	8–32	100	0	0
	Tetracycline	0.5	1	≤0.12 to >4	9.0	83.0	8.0
2022 (123)	Zoliflodacin	0.12	0.12	≤0.008–0.25	100	NA	NA
	Azithromycin	0.12	0.5	≤0.06 to >8	97.6	NA	NA
	Cefixime	0.015	0.03	≤0.002–0.12	96.8	3.2	0
	Ceftriaxone	0.008	0.015	≤0.002–0.03	100	0	0
	Ciprofloxacin	0.004	>2	0.001 to >2	64.2	0.8	35
	Gentamicin	8	8	1–16	NA	NA	NA
	Penicillin	0.5	>2	≤0.12 to >2	NA	89.4^*b*^	10.6
	Spectinomycin	16	16	≤4–32	100	0	0
	Tetracycline	1	>4	≤0.12 to >4	10.6	68.3	21.1

^*a*^ MIC interpretations were based on CLSI interpretive criteria except for zoliflodacin, which was based on the FDA susceptible-only breakpoint of ≤0.25 µg/mL (13).

^*b*^ Non-resistant because unable to separate susceptible from intermediate due to range tested.

^*c*^ I, percent intermediate; NA, not applicable; %R, percent resistant; %S, percent susceptible according to CLSI interpretive criteria (12).

All 322 *N. gonorrhoeae* isolates were susceptible to zoliflodacin, based on the FDA susceptibility breakpoint of ≤0.25 µg/mL (Table 1) (13). The zoliflodacin MIC values ranged from ≤0.008 to 0.25 µg/mL with MIC_50_ and MIC_90_ values both 0.12 µg/mL. The potency of zoliflodacin was consistent across years 2020 to 2022, where MIC_50/90_ values ranged from 0.06/0.12 µg/mL to 0.12/0.12 µg/mL (Tables 1 and 2). Activity of zoliflodacin was also comparable for isolates collected from both males and females (MIC_50/90_ values of 0.12/0.12 µg/mL and 0.06/0.12 µg/mL, respectively). Susceptibility to zoliflodacin remained high for isolates non-susceptible to azithromycin or tetracycline (Table 2). Importantly, zoliflodacin maintained activity against ciprofloxacin-resistant isolates with 100% of isolates with a zoliflodacin MIC ≤0.12 µg/mL. These data are consistent with biochemical studies demonstrating that, while fluoroquinolones and zoliflodacin each inhibit DNA gyrase, they have different mechanisms of inhibition and bind distinct sites on the DNA gyrase cleaved complex (8, 14–17).

**TABLE 2:** Cumulative frequency distributions of zoliflodacin MIC values against subsets of *Neisseria gonorrhoeae* isolates collected in the United States from 2020 to 2022

Subset (no. of isolates)	Value	Zoliflodacin MIC (µg/mL)^*a*^					
		≤0.008	0.015	0.03	0.06	0.12	0.25
All isolates (322)	Cumulative %	5.6	6.2	12.7	48.1	**97.2**	100
	Percent	5.6	0.6	6.5	35.4	49.1	2.8
	Number	18	2	21	114	158	9
Male (287)^*b*^	Cumulative %	6.3	6.6	13.6	46.3	**96.9**	100
	Percent	6.3	0.3	6.9	32.8	50.5	3.1
	Number	18	1	20	94	145	9
Female (26)^*b*^	Cumulative %	0	3.8	3.8	53.8	**100**	100
	Percent	0	3.8	0	50.0	46.2	0
	Number	0	1	0	13	12	0
Ciprofloxacin-R^*c*^ (113)	Cumulative %	5.3	6.2	18.6	74.3	**100**	100
	Percent	5.3	0.9	12.4	55.8	25.7	0
	Number	6	1	14	63	29	0
Azithromycin-NS^*d*^ (12)	Cumulative %	0	0	0	8.3	75.0	**100**
	Percent	0	0	0	8.3	66.7	25.0
	Number	0	0	0	1	8	3
Tetracycline-R^*e*^ (296)	Cumulative %	5.1	5.7	11.8	45.9	**97.0**	100
	Percent	5.1	0.7	6.1	34.1	51.0	3.0
	Number	15	2	18	101	151	9
2020 (99)	Cumulative %	7.1	8.1	14.1	50.5	**97.0**	100
	Percent	7.1	1.0	6.1	36.4	46.5	3.0
	Number	7	1	6	36	46	3
2021 (100)	Cumulative %	5.0	5.0	17.0	54.0	**100**	100
	Percent	5.0	0	12.0	37.0	46.0	0
	Number	5	0	12	37	46	0
2022 (123)	Cumulative %	4.9	5.7	8.1	41.5	**95.1**	100
	Percent	4.9	0.8	2.4	33.3	53.7	4.9
	Number	6	1	3	41	66	6

^*a*^ MIC_90_ is in boldface for each MIC distribution.

^*b*^ There were nine isolates where the sex of the patient was unspecified. These nine were not included in the distributions for isolates specified as from male or female patients.

^*c*^ Ciprofloxacin-resistant (MIC ≥ 1 µg/mL).

^*d*^ Azithromycin non-susceptible (MIC > 1 µg/mL).

^*e*^ Tetracycline resistant (MIC ≥ 2 µg/mL).

The activity of zoliflodacin was also measured against two ceftriaxone-resistant *N. gonorrhoeae* encoding *penA 60.001* alleles isolated in the United States in 2019 and 2022 that were acquired from the CDC Antimicrobial Resistance Isolate Bank (AR 1280 and 1281) (18). The zoliflodacin MICs were 0.06 and 0.25 µg/mL against these isolates, similar to other reports of zoliflodacin activity against ceftriaxone-non-susceptible isolates (19, 20).

The whole genome sequences of *N. gonorrhoeae* isolates from this study originated from the National Center for Biotechnology Information Bacterial Antimicrobial Resistance and Reference Gene Database (accession number PRJNA317462). The genomic relationship between the isolates and the 2024 WHO reference strains (*n* = 15), representing known susceptible and resistant phenotypes and genotypes for antibiotics used in the treatment of gonorrhea (21), was visualized by a phylogenetic tree (22). Figure 1 shows that the isolates represent a diverse set of genotypes across all 3 years (2020–2022) and contain 45 unique NG-STAR clonal complexes (23) and several strains that remain ungrouped and/or are novel in the NG-STAR database. The zoliflodacin MIC values vary across the entire phylogenetic tree, with little bias toward the year the strains were isolated, clonal complex, or susceptibility to ciprofloxacin.

**Fig 1:**
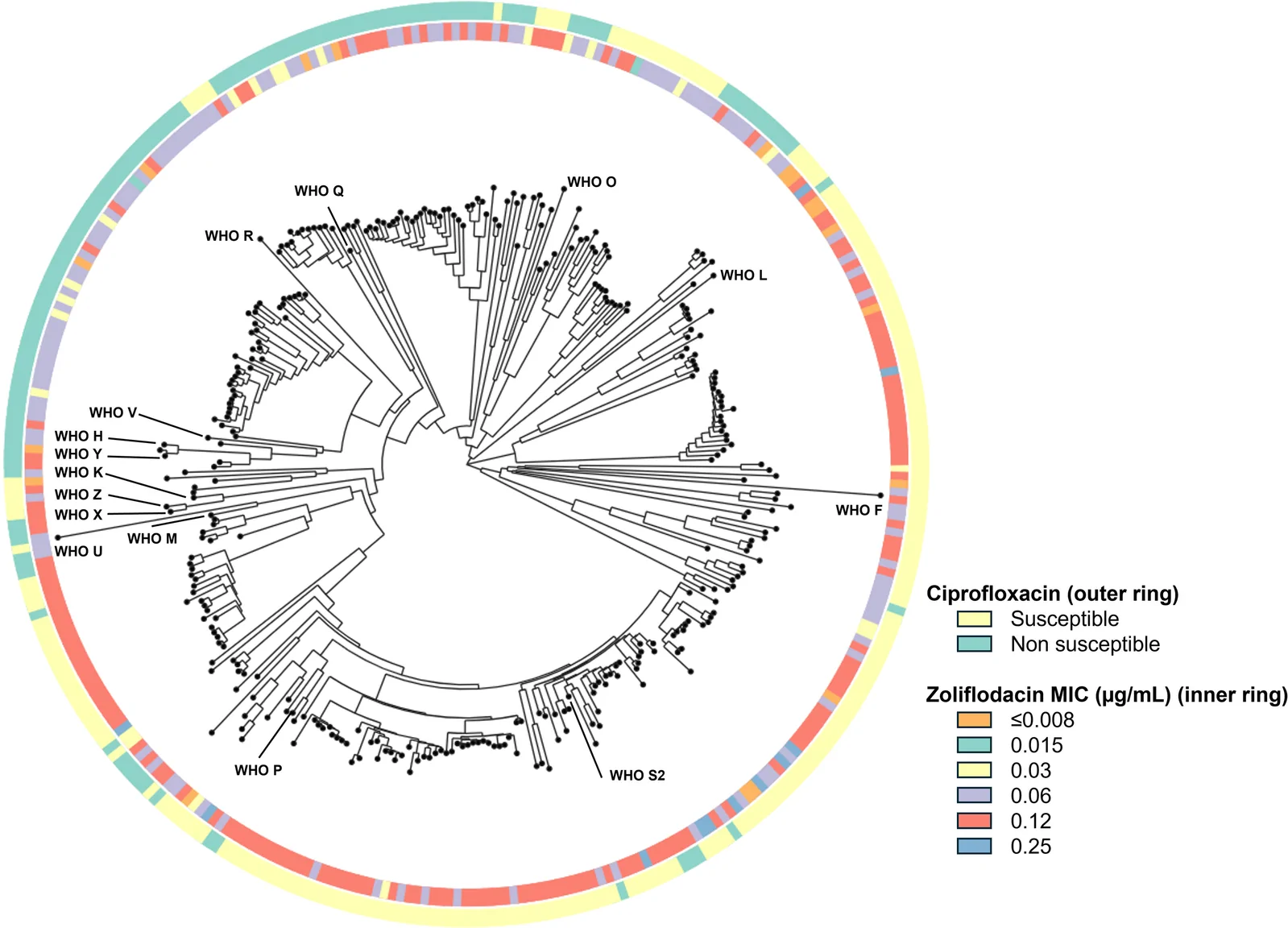
Circular phylogeny tree of 322 *N. gonorrhoeae* isolates from the United States and 15 WHO reference strains.

It should be noted that this study was conducted on isolates collected from patients in the United States, which may not represent the global population, and that *in vitro* activity does not necessarily predict clinical response. However, the data from this study are consistent with multiple reports of zoliflodacin *in vitro* activity against *N. gonorrhoeae* isolates from around the globe, including isolates from North America, Europe, Asia, and Africa (8, 9, 20, 24–27). Across these studies, zoliflodacin MIC_90_ values ranged from 0.06 to 0.25 µg/mL, similar to what was observed here. Future surveillance of zoliflodacin activity will be important as resistance to new treatments for gonorrhea have historically emerged rapidly.

In summary, zoliflodacin demonstrated potent *in vitro* activity against clinical *N. gonorrhoeae* isolates collected in 2020–2022 by the CDC across the United States. These results, together with prior studies, support zoliflodacin as an important option for the treatment of uncomplicated gonorrhea, including infections caused by antibiotic-resistant strains.
